# Prevalence of allergic rhinitis, related comorbidities and risk factors in schoolchildren

**DOI:** 10.1186/s13223-020-00495-1

**Published:** 2020-11-11

**Authors:** Monika Sultész, Alpár Horváth, Dávid Molnár, Gábor Katona, Györgyi Mezei, Andor Hirschberg, Gabriella Gálffy

**Affiliations:** 1 Department of Oto-Rhino-Laryngology, Heim Pál National Pediatric Institute, 86. Üllői street, Budapest, 1089 Hungary; 2 Pest County Pulmonology Hospital, 70. Munkácsy Mihály Street, Törökbálint, 2045 Hungary; 3 Medical Department of Chiesi Hungary Ltd, 2. Dunavirág street, Budapest, 1138 Hungary; 4 Department of Otorhinolaryngology and Head and Neck Surgery, Medical Centre, Hungarian Defence Forces, 109-111. Podmaniczky street, Budapest, 1062 Hungary; 5 Department of Anatomy, Histology and Embryology, Semmelweis University, 58 Tűzoltó street, Budapest, 1085 Hungary; 6 Department of Oto-Rhino-Laryngology, Heim Pál National Pediatric Institute, 86. Üllői street, Budapest, 1089 Hungary; 7 Division of Allergo-Pulmonology, 1st Department of Paediatrics, Semmelweis University, 53-54 Bókay János street, Budapest, 1083 Hungary; 8 Department of Oto-Rhino-Laryngology and Maxillo-Facial Surgery, Saint John’s Hospital, 1-3. Diós árok, Budapest, 1125 Hungary; 9 Department of Thoracic Surgery, Semmelweis University, 7-9 Ráth György street, Budapest, 1122 Hungary

**Keywords:** Allergic rhinitis, Related atopic diseases, Budapest, ISAAC, Prevalence, Risk factors, Schoolchildren

## Abstract

**Background:**

The study aimed to determine the prevalence and risk factors of allergic rhinitis and related comorbidities in school-age children in Budapest, capital of Hungary. Data and epidemiological studies on this disease are still limited.

**Methods:**

A cross sectional study was conducted in 21 representative and randomly selected primary schools in 2019. International Study of Asthma and Allergies in Childhood-based questionnaires (n = 6869) inquiring about prevalence and related risk factors of allergic rhinitis were distributed to all parents. The data were characterised with standard descriptive statistics: frequencies (percentages) and means for categorical and quantitative data, respectively.

**Results:**

3836 of the questionnaires (1857 M/1979F) were completed. The prevalence of current allergic rhinitis was 29.3% (1043), physician-diagnosed allergic rhinitis was 9.7% (373), cumulative allergic rhinitis was 36.2% (1289) and current allergic rhinoconjunctivitis was 16.2% (577). The presence of physician diagnosed atopic disease–asthma (p < 0.0001, OR = 4.398, 95% CI 3.356–5.807), food allergy (p < 0.0001, OR = 2.594, 95% CI 1.995–3.378), and eczema (p < 0.0001, OR = 1.899, 95% CI 1.568–2.300)-were significantly related to an increased risk of cumulative allergic rhinitis. Significant factors associated with allergic rhinitis include male gender (p < 0.0001), family history of atopy (p < 0.0001), frequent upper respiratory tract infections (p < 0.0001), tonsillectomy (p = 0.0054), antibiotics given in the first year of life (p < 0.0001), paracetamol given in the first year of life (p = 0.0038), long-lasting common infections caused by viruses and/or bacteria before the appearance of the allergy (p < 0.0001), consumption of drinks containing preservatives or colourants (p = 0.0023), duration of living in Budapest (p = 0.0386), smoking at home (p = 0.0218), smoking at home in the first year of life (p = 0.0048), birds at home (p = 0.0119), birds at home in the first year of life (p = 0.0052), visible mould in the bedroom (p = 0.0139), featherbedding (p = 0.0126), frequent or constant heavy-vehicle traffic (p = 0.0039), living in a weedy area (p < 0.0001) and living in the vicinity of an air-polluting factory or mine (p = 0.0128).

**Conclusions:**

The prevalence of allergic rhinoconjunctivitis in 6–12-year-old children in Budapest is higher than reported for most of the surrounding European countries. While asthma (OR = 4.398) is the most significant comorbidity, environmental factors such as birds at home in the first year of life (OR = 2.394) and living in a weedy area (OR = 1.640) seem to be the most important factors associated with AR. Strategies for preventive measures should be implemented.

*Trial registration number*: KUT-19/2019. The study was approved by the Ethics Committee at Heim Pál National Pediatric Institute,

## Background

Allergic rhinitis (AR) is one of the most common chronic disorders of the pediatric population. About 44–87% of patients with rhinitis may have a combination of allergic and nonallergic rhinitis [1]. Regardless of the high prevalence of AR in childhood, the disease is often underdiagnosed or undertreated. Untreated and undertreated AR deteriorates the quality of life of the child and his or her family. AR places a financial burden on the healthcare system, including direct and indirect costs. Due to an increase in the prevalence of allergic conditions in Western and developing countries, the reason of which was unknown, Asher and coworkers founded the International Study of Asthma and Allergies in Childhood (ISAAC) in 1991, which is a unique worldwide epidemiological research programme. In Phase I, using standardised and validated questionnaires for 6–7-year-old and 13–14-year-old schoolchildren, they estimated the prevalence of allergic diseases around the world [2]. Phase II was based on the findings of Phase I, but beyond the prevalence data, it also measured the possible etiological factors of asthma, rhinoconjunctivitis and eczema. After a 5–10 year interval, Phase III provided follow-up data in multiple centres worldwide [3]. According to this study, the prevalence of AR varied between 0.8 to 14.9% in 6–7-year olds and 1.4 to 39.7% in 13–14-year old children worldwide. Phase III of ISAAC examined the possible risk factors of AR with new questions. Genetic factors, family history of atopy and allergic diseases play an influential role in AR presentation. However, environmental factors and lifestyle had also been considered important in the disease.

In the ISAAC phase III research (2009) the Hungarian data (measured in 2003) were also published: the prevalence of AR symptoms was 12.9% in children aged 6–7-years and 17.1% in children aged 13–14 years [4]. To date, there has been a limited number of epidemiological studies in the population of 6–12-year old pupils in Hungary. AR presentation and manifestation might be affected by several factors, but their exact nature remains poorly understood.

The purpose of the article is to assess the prevalence of AR in 6–12-year-old schoolchildren, as well as to identify the risk factors associated with this disease. We also aimed to explore the relationship between AR and other allergic conditions.

## Methods

### Study design

**Fig. 1:**
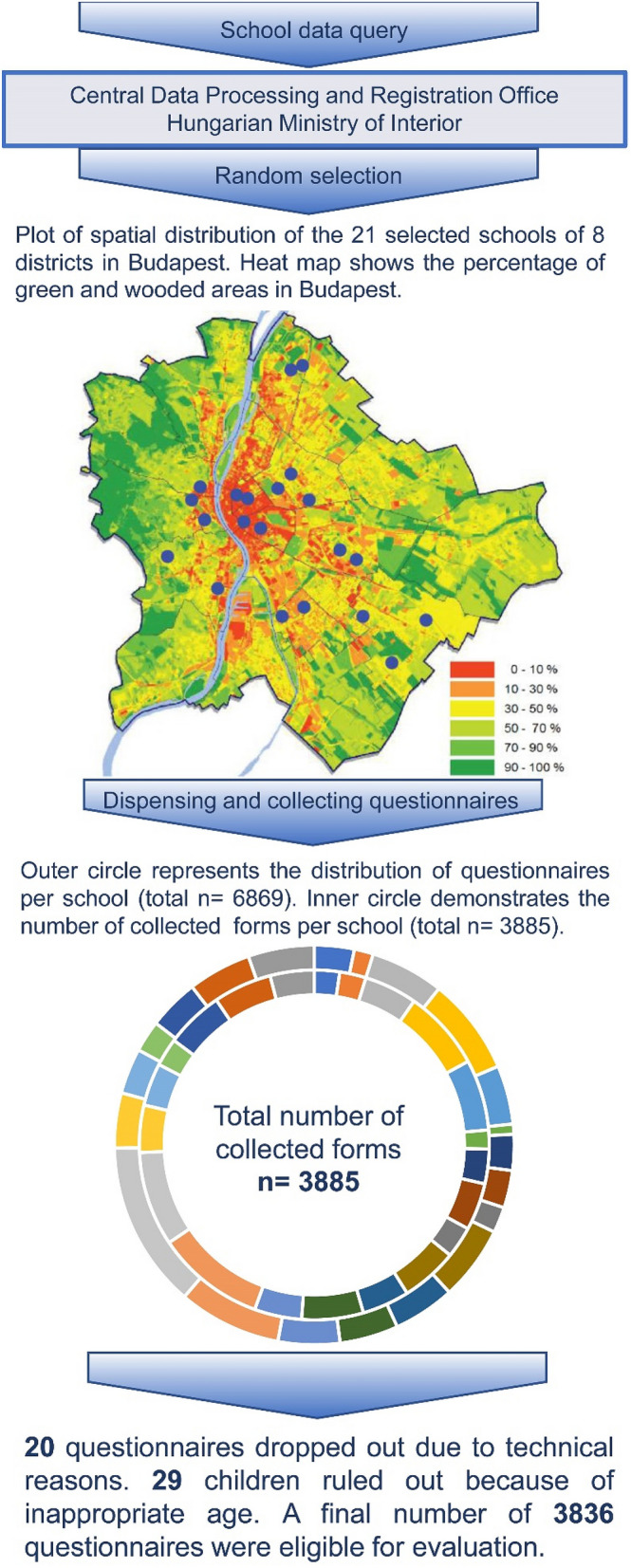
Schematic demonstration of data collection and preparation

This cross-sectional study was carried out in September 2019 in Budapest (Hungary). 6–12-year-old children were randomly selected from the representative 8 districts and 21 primary schools. The selection of schools was based on the district listings provided by Central Data Processing and Registration Office of the Hungarian Ministry of Interior. From 6869 distributed questionnaires 3885 were collected (response rate: 56,6%). 20 questionnaires were excluded due to technical reasons. 29 children were ruled out due to their age. Finally, the valid number of questionnaires were 3836 (Fig. 1). At the first teacher-parent meetings of the school year, the parents were asked to complete ISAAC-based questionnaires. The teachers gave detailed instructions before completion. The questionnaires were collected immediately after the teacher-parent meetings, or a week later at most.

### The questionnaire

The questionnaire consisted of two parts. In part one the Hungarian-translated version of the core questions of ISAAC Phase I and its methodology were used [4], which included questions posed to parents related to the prevalence of atopic diseases in their children; the information requested included:

- parental reports of physician-diagnosed allergic diseases (AR, conjunctivitis, asthma, eczema, food-allergy)
- parental reports of current symptoms of AR and conjunctivitis

The prevalence of diagnosed allergic disease was determined based on the responses to the question “Has your child been diagnosed with an allergic disease by a physician?” If the response was positive, the question, “What kind of allergic disease was it?” identified those pupils who suffered from eczema, food allergy or asthma.

The prevalence of “diagnosed AR” was determined based on the answers to the question “Has your child had allergic rhinitis diagnosed by a physician?” The parents of those pupils, who answered yes to this (above mentioned) question, did not get questions about current AR symptoms. “Current AR” group consists of pupils who had not been diagnosed with AR by a physician, but whose parents gave a positive response to the following question: “In the past 12 months, has your child had a problem with sneezing, or a runny, or a blocked nose when he/she did not have a cold or the flu?” In this way, there was no overlap between the two groups. The third question related to allergic rhinoconjunctivitis “In the past 12 months, has this nose problem been accompanied by itchy-watery eyes?” A positive response supports the presence of current allergic rhinoconjunctivitis. Cumulative AR was calculated by taking the number of patients with current AR, adding the number of patients with physician diagnosed AR, and subtracting those who overlapped. Parents were also asked whether rhinitis symptoms interfered with their child’s daily activities or disturbed their child’s sleep in the past 12 months.

The second part of the questionnaire included questions regarding associated environmental factors for cumulative AR symptoms, for example "Do you have animals at home?" or "Is there an extensive weedy area in the vicinity of the flat (within 500 m)?" The detailed questionnaire can be found in the Additional file 1.

### Statistical analysis

The data were characterised with standard descriptive statistics: frequencies (percentages) and means for categorical and quantitative data, respectively.

Binomial logistic regression was used to compare frequencies, and the t-test was used to compare means of groups. Results were considered statistically significant at p < 0.05.

In case of categorical variables, odds ratios (OR) and 95% confidence intervals (95% CI) were calculated in order to establish how much more likely it was that someone who had the risk factor would develop allergy compared with someone who did not have it.

Prevalence estimates were calculated by dividing positive responses to the given question by the total number of completed questionnaires.

Percentages were calculated by dividing the frequency by the total number of observations, excluding missing answers, and then multiplying by 100.

All analyses were performed with the R 3.6.2 statistical program software (R for Windows 3.4.2 (R Core Team 2017, R: A language and environment for statistical computing. R Foundation for Statistical Computing, Vienna, Austria. URL https://www.R-project.org/).

## Results

### Prevalence data of allergic rhinitis and atopic diseases

**Fig. 2:**
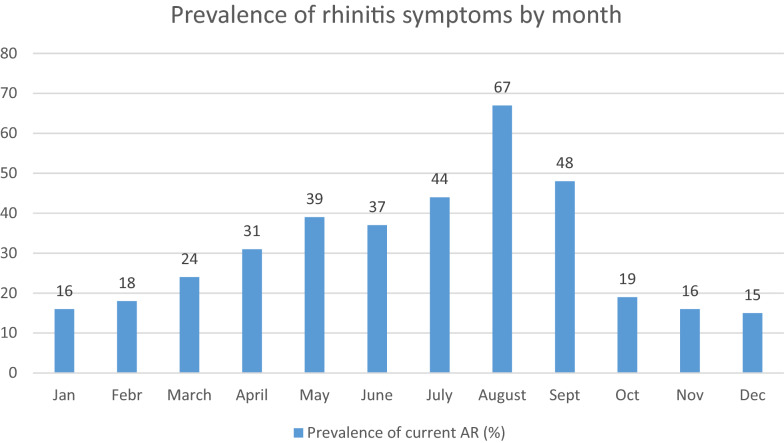
Monthly distribution of rhinitis symptoms in children in the last 12 months

In the study boy to girl ratio was 48.4% to 51.6%, respectively. For the cumulative AR group, this ratio was reversed this ratio was reversed (55%:45%). Of 3836 children 1206 (31.4%) children reported having been diagnosed by a physician with an atopic disease 491 (12.8%) had eczema, 242 (6.3%) had a food allergy, 248 (6.5%) had asthma, 373 (9.7%) had AR and 307 (8.0%) had allergic conjunctivitis (Table 1). Prevalence data for current AR was 1043 (29.3%), physician-diagnosed AR was 373 (9.7%), and symptom-based cumulative AR was 1289 (36.2%) (Table 2). The prevalence of current rhinoconjunctivitis was 577 (16.2%); these pupils had current rhinitis symptoms accompanied by current conjunctivitis symptoms. Monthly distribution showing a seasonal pattern with a peak between July and September (Fig. 2). It is shown in Table 3 that from all the examined patients 1043 (29.3%) suffered from current AR. Among these children, there were 33 (3.2%), whose daily activity was severely disturbed by the nasal symptoms. Two thirds of these children woke up once or more weekly during the nights. Conversely, no more than one third of the children (148) with mild nasal symptoms, woke up once or more weekly during the night. From the 1043 children suffering from current AR about one third (291) had disturbed sleep due to nasal symptoms.

**Table 1:** Prevalence and distribution of physician-diagnosed specific allergic diseases (eczema, food allergy, asthma, allergic rhinitis and allergic conjunctivitis)

	Eczema		Food allergy		Asthma		Allergic rhinitis		Allergic conjunctivitis	
	N	%	N	%	N	%	N	%	N	%
Yes	491	12.8	242	6.3	248	6.5	373	9.7	307	8.0
No	3345	87.2	3594	93.7	3588	93.5	3463	90.3	3529	92.0
Total	3836 (100%)

**Table 2:** Prevalence of allergic rhinitis in the study

Type of AR	Number of cases	Percentage	Valid percentage
Current AR			
No	2521	65.7	70.7
* Yes*	1043	27.2	29.3
Total	3564	92.9	100
* NA*	272	7.1	
Total	3836	100	
Physician- diagnosed AR			
No	3463	90.3	90.3
Yes	373	9.7	9.7
Total	3836	100	100
Cumulative AR			
No	2275	59.3	63.8
* Yes*	*1289*	33.6	36.2
Total	3564	92.9	100
* NA*	272	7.1	
Total	3836	100	

The "*Percentage*" column represents the percentage of all cases, including the patient with missing data. "*Valid percentage*" is the percent when missing data are excluded from the calculations

*AR* allergic rhinitis, *NA* not available

**Table 3:** Relationship between sleep disturbances and daily nasal symptoms among children suffering from current allergic rhinitis

Frequency of disturbed sleeping due to AR-related nasal symptoms in the last 12 months	Interference with daily activity due to AR-related nasal symptoms in the last 12 months				
	A lot (%)	A moderate amount (%)	A little (%)	Not at all (%)	Total (marginal)
1 or more times per week	21 (63.6)	96 (63.6)	148 (30.1)	26 (7.1)	291 (27.9)
< 1 more per week	6 (18.2)	33 (21.9)	162 (32.9)	65 (17.7)	266 (25.5)
Never	6 (18.2)	22 (14.6)	182 (37.0)	276 (75.2)	486 (46.6)
Total (marginal)	33 (3.2)	151 (14.5)	492 (47.2)	367 (35.2)	1043 (100.0)

The daily nasal symptoms are represented in the columns, while nose problems during the nights are shown in the rows

*AR* allergic rhinitis

### Risk factors for cumulative allergic rhinitis

Tables 4 and 5 present the relationship between the risk factors and the prevalence of cumulative AR. Boys had significantly higher chance to develop cumulative AR symptoms than girls. The prevalence of physician-diagnosed asthma (p < 0.0001, OR = 4.398, 95% CI 3.356–5.807), physician-diagnosed food allergy (p < 0.0001, OR = 2.594, 95% CI 1.995–3.378) and physician-diagnosed eczema (p < 0.0001, OR = 1.899, 95% CI 1.568–2.300) were significantly associated with an increased risk of cumulative AR. Family history of atopy, frequent upper respiratory tract infections, tonsillectomy, antibiotics or paracetamol given in the first year of life, long-lasting common infections caused by viruses and/or bacteria before the appearance of the allergy, consumption of drinks containing preservatives or colourants, duration of living in Budapest, smoking at home, smoking at home in the first year of life, birds at home, birds at home in the first year of life, visible mould in the bedroom, featherbedding, frequent or constant heavy-vehicle traffic, living in a weedy area and living in the vicinity of an air-polluting factory or mine increased the risk of the development of AR. Adenoidectomy, living in a house with prefabricated concrete walls and living in a green area were not correlated with cumulative AR.

**Table 4:** Factors affecting cumulative allergic rhinitis symptoms

Factors	Cumulative allergic rhinitis symptoms n (%)	p-value	OR	CI
Sex				
M (n = 1857)	713 (38.4)	< 0.0001	0.672	0.587–0.768*
F (n = 1979)	584 (29.5)			
Physician-diagnosed asthma				
Yes (n = 248)	166 (66.94)	< 0.0001	4.398	3.356–5.807*
No (n = 3588)	1131 (31.52)			
Physician-diagnosed food allergy				
Yes (n = 242)	134 (55.4)	< 0.0001	2.594	1.995–3.378*
No (n = 3594)	1163 (32.4)			
Physician-diagnosed eczema				
Yes (n = 491)	231 (47.1)	< 0.0001	1.899	1.568–2.300*
No (n = 3345)	1066 (31.9)			
Family history of atopy				
Yes (n = 2483)	999 (40.2)	< 0.0001	2.380	2.050–2.776*
No (n = 1353)	298 (22.0)			
Frequent upper respiratory tract infections				
Yes (n = 872)	482 (55.3)	< 0.0001	3.259	2.789–3.811*
No (n = 2964)	815 (27.5)			
Tonsillectomy				
Yes (n = 138)	62 (44.9)	0.0054	1.627	1.152–2.289*
No (n = 3698)	1235 (33.4)			
Adenoidectomy				
Yes (n = 523)	195 (37.3)	0.0712	1.193	0.938–1.443
No (n = 3313)	1102 (33.3)			
Antibiotics given in the first year of life				
Yes (n = 1075)	418 (38.9)	< 0.0001	1.362	1.176–1.577*
No (n = 2761)	879 (31.8)			
Paracetamol given in the first year of life				
Yes (n = 1344)	495 (36.8)	0.0038	1.229	1.069–1.412*
No (n = 2494)	802 (32.2)			
Long-lasting common infections caused by viruses and/or bacteria disease before the appearance of the allergy				
Yes (n = 122)	98 (80.3)	< 0.0001	8.565	5.549–13.754*
No (n = 3714)	1199 (32.3)			
Consumption of drinks containing preservatives or colorants				
Yes (n = 1550)	568 (36.7)	0.0023	1.235	1.079–1.415*
No (n = 2286)	729 (31.9)			

p < 0.05 considered significant

*OR* odds ratio, *CI* confidence interval

*significant association

**Table 5:** Environmental factors affecting cumulative allergic rhinitis symptoms

Factors	Cumulative allergic rhinitis symptoms n (%)	p-value	OR	CI
Duration of living in Budapest				
5 years or more (n = 3366)	1158 (34.4)	0.0386	1.249	1.014–1.545^a^
0–less than 5 years (n = 470)	139 (29.6)			
Smoking at home				
Yes (n = 370)	145 (39.2)	0.0218	1.295	1.037–1.612^a^
No (n = 3466)	1152 (33.2)			
Smoking at home in the first year of life				
Yes (n = 325)	133 (40.9)	0.0048	1.397	1.106–1.760^a^
No (n = 3511)	1164 (33.1)			
Birds at home				
Yes (n = 111)	50 (45.05)	0.0119	1.629	1.110–2.379^a^
No (n = 3725)	1247 (33.5)			
Birds at home in the first year of life				
Yes (n = 42)	23 (54.8)	0.0052	2.394	1.301–4.460^a^
No (n = 3794)	1274 (33.6)			
Visible mould in the bedroom				
Yes (n = 122)	54 (44.3)	0.0139	1.579	1.094–2.268^a^
No (n = 3714)	1243 (33.5)			
Featherbedding				
Yes (n = 546)	159 (29.1)	0.0126	0.777	0.636–0.945^a^
No (n = 3290)	1138 (34.6)			
Living in a house with prefabricated concrete walls				
Yes (n = 1013)	367 (36.2)	0.0582	1.156	0.995–1.343
No (n = 2823)	930 (32.9)			
Heavy-vehicle traffic frequent or constant				
Yes (n = 2483)	880 (35.4)	0.0039	1.232	1.070–1.421^a^
No (n = 1353)	417 (30.8)			
Living in a green area				
Yes (n = 1570)	513 (32.7)	0.2159	0.917	0.801–1.051
No (n = 2266)	784 (34.6)			
Living in a weedy area				
Yes (n = 1666)	668 (40.1)	< 0.0001	1.640	1.433–1.877^a^
No (n = 2170)	629 (29.0)			
Living not far from an air-polluting factory or mine				
Yes (n = 689)	261 (37.9)	0.0128	1.243	1.047–1.473^a^
No (n = 3147)	1036 (32.9)			

*OR* odds ratio, *CI* confidence interval

p < 0.05 considered significant

^a^significant association

## Discussion

### Epidemiology of allergic rhinitis

This epidemiological study investigated the prevalence and risk factors of AR using an ISAAC questionnaire in primary schoolchildren in Budapest (Hungary). Of the examined primary schoolchildren, 31.4% (n = 1206) were found to suffer from a diagnosed atopic disease. The prevalence of this cohort was 12.8% (n = 491) for physician-diagnosed eczema, 6.3% (N = 242) for physician-diagnosed food allergy, 6.5% (n = 248) for physician-diagnosed asthma, 9.7% (373) for physician-diagnosed AR and 8.0% (n = 307) for physician-diagnosed allergic conjunctivitis (Table 1). The prevalence of physician-diagnosed allergic diseases in our survey was lower than that in Katowice (2019) in 6–9-year-old children (physician-diagnosed asthma 6.6%, physician-diagnosed AR 22.1%, physician-diagnosed eczema 20.5%). However, it was higher than that in Eastern Croatia (2008) in 10–11-year-old children (physician-diagnosed asthma 4.1%, physician-diagnosed AR 6.3%, physician-diagnosed eczema 11.8%) [5, 6].

We demonstrated a relatively high prevalence of current AR, current rhinoconjunctivitis, physician-diagnosed AR and cumulative AR. Taking the number of patients with current AR, and adding the number of patients with physician diagnosed AR, and then subtracting those who overlapped gave the number of patients with cumulative AR (Table 2), probably related to environmental factors (air pollution, high pollen concentration). The Global Phase III of ISAAC analyses revealed that the 12-month prevalence for rhinoconjunctivitis was 8.5% in the 6–7-year-old children, and 14.6% in the 13–14-year-old [4]. In 2003 Hungary connected to ISAAC Phase III with two regions (East and Central) and examined nearly 10,000 children. The centre of the Central region was Svábhegy (Budapest), where AR for 6–7 year olds and 13–14 year olds were 5.3% and 9.5% [4]. Based on our study, which was performed among 6–12-year-old students using the ISAAC methods, the prevalence of current allergic rhinoconjunctivitis is estimated at 16.2%. Thus, it can be interpreted that since 2003 the prevalence of current allergic rhinoconjunctivitis has increased in our capital, and it is higher than the average global ISAAC data colleted in 2002–2003 [4]. Comparing our prevalence result with that of surrounding countries in Europe carried out according to the ISAAC protocol in the previous years – Eastern Croatia 9.9%; Ukraine, Kyev (2018) 13.4% in 13–14-year-old children and 10.6% in 6–7-year-old children; Poland, Katowice 25.8%–we can conclude that the prevalence of current rhinoconjunctivitis in Budapest is higher, except Katowice [5–7].

Our statistical analysis results showed that the prevalence of severe current AR-related symptoms was 3.2% regarding the last 12 months (Table 3). The nasal symptoms of these children largely interfered with their daily activities. 27.9 per cent of the pupils suffering from current AR had disturbed sleep due to nasal problems. According to an extensive survey from the USA, nasal congestion affects most patients with AR and has a significant impact on the quality of life [8]. In our study, the nasal blockage interfered with the pupils’ daily activity and disturbed their sleep more than any other symptoms (sneezing, rhinorrhoea, itchy nose). Figure 2 depicts the changes in the appearance of nasal symptoms of children with current AR depending on the seasons in Budapest. The appearance of current AR symptoms was as low as 15% in January. This gradually increased to 18% in February, to 24% in March and was as high as 39% in May. A marked elevation of the symptom appearance was measured from June (37%) to the end of October (19%), with the peak in August (67%). From November to the end of the year the appearance of the symptoms was around 15–16%. In Hungary, there are six relevant groups among the most common allergenic plants: birch tree (Betulaceae), grasses (poaceae), Asteraceae (Compositae), the olive family (oleaceae), stinging nettle family (urticaceae), gymnosperm plants (gymnospermae). From all of the plants that present in pollen-spreading season, each one is a potent allergen, and their occurrence is frequent. Flowering interval starts in January and ends in October in Hungary. We can distinguish three main periods for pollen-spreading, the first one is that of trees, which are flowering between January and April, followed by the season of grasses, from May to July, with the weed/ragweed season in the end, from July to the end of October. The Pannonian Biogeographical Region (Carpathian Basin), especially Hungary, is the most affected area in Europe regarding ragweed (*Ambrosia artemisiifolia*) the most allergenic wind-pollinated plant.

### Risk factors for cumulative allergic rhinitis

Our study showed significant male predominance for the development of AR in the examined age group. This finding is similar to other studies [9, 10]. Before the onset of puberty, boys outnumber girls in terms of prevalence, but the trend reverses after puberty. It has been proposed that the difference is caused by a real impact of female sex hormones on disease development. Physician-diagnosed asthma, food allergy and eczema were shown to be significant risk factors for the development of cumulative AR in our study. One suggested mechanism for the explanation of the connection between atopic disorders and AR is atopic march [11]. According to this theory, the atopic diseases progress from atopic dermatitis to asthma and, subsequently, to AR. A retrospective cohort study from the USA [12] reported that patients with multiple food allergies had increased risk of developing respiratory allergies. A population-based prospective study [13] confirmed that early eczema affects later development of respiratory allergies. Genetic factors, such as filaggrin gene mutation account for skin barrier dysfunction. A dysfunctional skin barrier serves as a site for allergic sensitisation to antigens and colonisation of bacterial superantigens. This triggers Th2 immunity and the development of atopic diseases. Following the literature, a family history of atopy was found as a strong significant risk factor for AR. Birth cohort studies [14, 15] have shown that a family history of atopy is a key risk factor associated with increased risk for AR expression. In the present study, we found a significant relationship between AR and missing palatinal tonsils due to tonsillectomy. Tonsils are parts of the immune system, their activity in early-life is important, while this time the development of the immune system is sensitive. Tonsillectomy may cause a change not only in the humoral but also in the cellular immune system. Per the hygiene hypothesis, a decrease in the immune response can cause an increased risk of the development of atopic diseases [16].

The survey did not detect a link between adenoidectomy, living in a house with prefabricated concrete walls, living in a green area and the occurrence of AR. Frequent upper respiratory tract infections (URTI) were found to be a significant risk factor for AR symptoms in the present study. An immunological research paper emphasised that virus-induced interferon-alpha production is decreased in children with an atopic phenotype [17]. This decline in innate immune antiviral responses may make them susceptible to developing AR, which results from frequent URTIs. The data presented in this article show that the intake of antibiotics in infancy increased the risk of AR in children aged 6–12 years. The first year of life represents a critical period of immune development, and gut microbiota plays an important role. The antibiotic insult may lead to gut dysbiosis, which can cause early disruptions in the regulation of the immune system, and this may affect the development of AR [18]. We found a positive relationship between the consumption of paracetamol in the first year of life and the prevalence of cumulative AR. Paracetamol is a potential source of oxidative stress, as its use decreases the level of the antioxidant glutathione. As a result of this, the ability to resist oxidative stress decreases. Th2-mediated immunological response may be up-regulated [19, 20].

Our results revealed that long-lasting common infections caused by viruses and/or bacteria before the appearance of the allergy might increase the risk of the development of AR. The explanation is very similar as in the case of frequent URTIs. Children with atopic sensitisation have increased risk to the development of AR when experiencing serious virus illnesses. The reason is the decreased virus-induced interferon-alpha production mentioned above [17]. Consumption of drinks containing preservatives or colourants is significantly associated with AR symptoms in this research. In susceptible individuals, the frequency and intensity of allergic responses to antioxidants added in high doses as preservatives and colourants may play a major role. Th2-type immune response and allergy development can be increased [21].

We observed an increased risk of AR among individuals living five or more years in Budapest, compared with those who were living in the capital for less than 5 years. The difference was statistically significant. Our results also confirm that living near an air-polluting factory or mine is a risk factor associated with AR. As a result of transport emissions and domestic heating Budapest is one of the most polluted capital cities in Europe. In the city both particle pollution (PM_10_) and nitrogen dioxide (NO_2_) pollution are present. The major source of particle pollution is co-firing, but people burn textiles, plastic and other household wastes, too. The most common factory air pollutants are greenhouse gases from the burning of fossil fuels: carbon mono-oxid (CO), sulfur dioxide (SO_2_) and ozone (O_3_). Factories, particularly through the use of large industrial air conditioners, can also release destructive gases. Animal factories produce gases like methane, ammonia and others that lower air quality and are harmful to health. Air pollution in coal mines is mainly due to the fugitive emissions of particulate matter (SPM, RPM) and gases including methane, sulphur dioxide, oxides of nitrogen and carbon mono-oxide. When organic matter, such as diesel fuel, coal, wood or tobacco undergoes incomplete combustion, polycyclic aromatic hydrocarbons (PAHs) form. These are highly toxic compounds, characterised by fused aromatic rings. Recent research [22] provided evidence on the role of PAH exposure in the debase respiratory allergic diseases by inducing T cell changes at the epigenetic level leading to impairment of cellular and humoral immunity. A multicentre cross-sectional study from China provided a correlation of NO2, SO2, PM10, and PM2.5 levels and the risk of AR [23]. A Hungarian study demonstrated that smaller and fragmented pollens are more harmful than the large one, and may cause respiratory allergies [24]. Air pollution has a role in the fragmentation of pollens. We found an increased risk of AR among individuals who are exposed to smoke (smoking at home in the first year of life and smoking at home). Several published studies reported similar relationships. A recent study emphasised that tobacco smoke exposure facilitates sensitivity to allergens in children, and this can be measured by serum IgE level and skin test. In children exposed to parental smoke at home, the immunoregulatory mechanisms can increase the tendency for allergy [25]. Pet ownership is fashionable in our capital. A statistically significant correlation was noted between children with AR and keeping birds at home in the first year of life and later. The AR symptoms can be triggered by bird feather and the feather mites, which are also clinically-relevant allergens [26]. Visible mould in the bedroom was positively and statistically significantly associated with AR symptoms in our study. A recent article, which collected the results of systematic reviews and recent longitudinal studies [27], demonstrated sufficient evidence for an association between mould exposure and risk of development of AR. In this study, featherbedding correlated significantly with the prevalence of cumulative AR. True feather allergy is very rare. Some allergens can get trapped in the feathers, dust mites being the most common of them [28]. Animal dander, mould spores, and pollen can also be present. In agreement with other investigators, we found a positive relationship between AR symptoms and the frequency of truck traffic near home. Global data [29] have also provided evidence on the role of diesel particles that may enhance allergic sensitisation to common inhalant allergens. The risk of developing AR was strongly and significantly associated with living in a weedy area. In Hungary, common ragweed *Ambrosia artemisiifolia* is the most important cause of allergy-associated respiratory complaints. The Hungarian Great Plain is among the highest diurnal and annual ragweed pollen counts per m^3^ of air worldwide [30]. In Hungary, the flowering of *A. artemisiifolia* starts in the second half of July and ends in October (or with the onset of frost). The peak pollen season occurs from mid-August to mid-September. The timing of pollination is dependent on meteorological parameters. Because of global warming, the flowering season of ragweed has likely been extended. In Budapest, the level of atmospheric CO_2_ is often high, and this may also influence the growth and pollen production of the plant [30].

## Conclusions

The present cross-sectional epidemiologic study demonstrated that the prevalence of current allergic rhinoconjunctivitis (16.2%) in primary school children had increased in Budapest in the past 17 years, and it is higher than reported for most of the surrounding European countries. The proportion of children within the current AR group suffering from severe nasal symptoms is 3,2%. The majority of these patients have disturbed sleep due to their rhinitis symptoms, mainly in August. We found a strong association between atopic diseases asthma, eczema, food allergy and the presence of allergic rhinitis. Environmental factors may be responsible for the change in the prevalence data. The factors that are most likely to increase the chance of rhinitis are birds at home in the first year of life and living in a weedy area. Most patients have symptoms when ragweed (*Ambrosia artemisiifolia*) blooms.

Preventive measures (monitor programmes, eradication campaigns, prevention practices) should be taken both indoors and outdoors in order to stop the aggressive spread of ragweed and to inhibit its growth.

## Supplementary information

**Additional file 1.** Allergic rhinitis and asthma questionnaire for 6- to 12-year-olds.

## Data Availability

The datasets during and/or analysed during the current study available from the corresponding author on reasonable request.
