# Supplementary material for: Prevalence of allergic rhinitis, related comorbidities and risk factors in schoolchildren
Source: Allergy Asthma Clin Immunol. 2020 Nov 11;16:98. doi: 10.1186/s13223-020-00495-1 (PMC7661153; doi:10.1186/s13223-020-00495-1)
Supplement: Supplementary file 1 — Additional file 1. Allergic rhinitis and asthma questionnaire for 6- to 12-year-olds. [file 13223_2020_495_MOESM1_ESM.docx]

Allergic rhinitis and asthma questionnaire for 6- to 12-year-olds

1. Is your child a: boy girl
2. Child’s date of birth: month ……….. year…………
3. The initials of your child’s name: …………………….
4. Child’s height: ………………………cm
5. Child’s bodyweight: …………………………….kg
6. The zipcode of your child’s home: ………………………
7. If in the previous 3 years the child lived in another place for at least for one year as his/her customary place of residence, the zipcode of that place: …………………
8. Has your child had doctor-diagnosed atopic disease (more than one answer is possible)? yes - no
9. What kind of physician diagnosed allergic disease has your child have (more than one answer is possible)?

eczema food allergy asthma allergic rhinitis allergic conjunctivitis

1. Has your child ever had doctor-diagnosed allergic rhinitis? Yes No

IF YOU ANSWERED "NO" PLEASE SKIP TO QUESTION 16

1. Has your child ever had a problem with sneezing, or a runny, or blocked nose when he/she did not have a cold or flu? Yes No
2. In the past 12 months, has your child had a problem with sneezing, or a runny or blocked nose when he/she did not have a cold or the flu? Yes No
3. In the past 12 months, has this nose problem been accompanied by itchy-watery eyes? Yes No
4. In which of the past 12 months did this nose problem occur? (please tick any which apply)

January [ ] February [ ] March [ ] April [ ] May [ ] June [ ] July [ ]

August [ ] September [ ] October [ ] November [ ] December [ ]

1. In the past 12 months, how much did this nose problem interfere with your child’s daily activities?

Not at all [ ] A little [ ] A moderate amount [ ] A lot [ ]

1. In the last 12 months, how often, on average, has your child’s sleep been disturbed due to this nose problem?

never woken [ ]

less than one night per week woken [ ]

one or more night per week woken [ ]

1. Has your child ever had wheezing or whistling in the chest at any time in the past?

Yes No

1. Has your child had wheezing or whistling in the chest in the last 12 months?

Yes No

1. How many attacks of wheezing has your child had in the last 12 months?

None 1 to 3 4 to 12 More than 12

1. In the last 12 months, how often, on average, has your child’s sleep been disturbed due to wheezing?

Never woken with wheezing [ ]

Less than one night per week [ ]

One or more nights per week [ ]

1. In the last 12 months, has wheezing ever been severe enough to limit your child’s speech to only one or two words at a time between breaths? Yes No
2. Has your child ever had asthma? Yes No
3. In the last 12 months, has your child’s chest sounded wheezy during or after exercise?

Yes No

1. In the last 12 months, has your child had a dry cough at night, apart from a cough associated with a cold or chest infection?

Yes No

1. Has your child had asthma diagnosed by a physician?

Yes No

1. Has the mother of the child ever had (more than one answer is possible) a. asthma b. eczema c. allergic rhinitis
2. Has the father of the child ever had (more than one answer is possible) a. asthma b. eczema c. allergic rhinitis

27. Has the brother/sister of the child ever had (more than one answer is possible)

a. asthma b. eczema c. allergic rhinitis

1. What type of building are you living in?

in a house with prefabricated concrete walls

in a brick house

in a detached house surrounded by a garden

in a log cabin

1. What can be found in the bedroom (more than one answer is possible)

wall-to-wall carpet

linoleum

parquet

tapestry

visible mould

plant

1. Does the child sleep in feather bedding? Yes No
2. Is heavy-vehicle traffic frequent or constant in the vicinity of the flat (within 500 metres)?
3. no b. rare c. regular
4. Is there am extensive weedy area in the vicinity of the flat (within 500 metres)?

Yes No

1. Is there an air-polluting factory or mine in the vicinity of the flat (within 1 km)?

Yes No

1. Do you have animals at home (more than one answer is possible)?

dog cat rodent bird

1. Did you have animals at home in the child’s first year of life (more than one answer is possible)?

dog cat rodent bird

1. Do you smoke in your home? Yes No
2. Did you smoke in your home in the child’s first year of life? Yes No
3. Does the child’s father smoke? Yes No
4. Does the child’s mother smoke? Yes No
5. Duration of the child’s living in Budapest? 5 years or more less than 5 years
6. Has your child have regular occurrence of frequent upper respiratory tract infections (more than 4 times yearly)? Yes No
7. Was your child given antibiotics in the first year of life? Yes No
8. Was your child given Paracetamol in the first year of life? Yes No
9. Did your child have long-lasting disease before the appearance of his/her allergy? Yes No
10. Has your child had adenoidectomy? Yes No
11. Has your child had tonsillectomy? Yes No
12. Did your child consume drinks containing preservatives or colorants in the last 12 months? Yes No
